# The Molecular and Antigenic Tissue Impact of Viral Infections on Liver Transplant Patients with Neonatal Hepatitis

**Published:** 2011-08-01

**Authors:** R. Yaghobi, B. Geramizadeh, S. Zamani, M. Rahsaz, N. Azarpira, M. H. Karimi, M. Ayatolahi, M. Hossein Aghdai, S. Nikeghbalian, A. Bahador, H. Salahi, S. A. Malek-Hosseini

**Affiliations:** 1 *Shiraz Transplant Research Center, Shiraz University of Medical Sciences, Shiraz, Iran,*; 2 *Organ Transplant Center, Shiraz University of Medical Sciences, Shiraz, Iran.*

**Keywords:** Neonatal hepatitis, Viral infections, PCR, IHC

## Abstract

Background: Pathogenesis of neonatal hepatitis relates to various underlying causes including viral infections. Both hepatotropic and non-hepatotropic viruses may induce liver failures in infants before birth, during delivery, or shortly after birth.

Objectives: The tissue impact of HCMV, HSV, HBV, HCV, and rotavirus and adenovirus infections was evaluated in studied infants with neonatal hepatitis.

Methods: The history of viral infections was analyzed in paraffin-embedded biopsy and autopsy tissues of 22 infants with neonatal hepatitis between years 1996 and 2007, retrospectively. The tissue molecular presentation of HBV, HCV, HCMV, HSV, adenovirus, and rotavirus was evaluated by different qualitative simple and nested PCR and RT-PCR protocols. Immunohistochemistry (IHC) method was used for studying the antigenic prevalence of HSV-1, 2; HBV, HCMV and adenovirus infections. Also the laboratory liver indices of all patients with neonatal hepatitis were analyzed.

Results: The HBV and HSV genomes were detected in 3 (14%) of 22 infants. The rotavirus and HCV-RNA and also the HCMV-DNA were detected separately in 1 (4%) of 26 paraffin-embedded autopsy and biopsy tissues. The HBV and HSV-1 specific antigens were separately diagnosed in 1 (4%) of 26 neonatal samples by IHC protocols. Also the HSV-2 antigen was seen in 5 (23%) of 22 liver autopsy and biopsy specimens. Co-infections with HCMV, HSV, HBV, HCV, and rotavirus were detected in these infants with hepatitis.

Conclusion: Diagnosis of single and mixed molecular and antigenic traces of HCMV, HSV, HBV, HCV and rotavirus underlines the etiologic role of these viruses in clinical pathogenesis of neonatal hepatitis.

## INTRODUCTION

After the first introduction of neonatal hepatitis terminology by Craig and Landing [1], study about unknown causes of liver dysfunction presented in infants aged less three months with conjugated hyperbilirubinemia, became important for many investigators in the field of pediatric hepatology [2-4]. Among various possible inducers of neonatal hepatitis, infectivity of the microorganism, especially viral infections, has crucial role for potential spreading of the organism—trans-placenta, intrapartum, and by lactation [5-7]. Hepatotropic viruses and viruses that indirectly, sporadically, and/or rarely infect the liver of infants are the main presumed infective causes of neonatal hepatitis [8-10]. Human cytomegalovirus (HCMV) with an annual infection rate of 1%–2% of worldwide newborns, is one of the most possible viral causes of hepatic dysfunction. Despite detection of HCMV in the epithelium of bile duct of neonates, this virus can only cause mild and uncommon infantile liver failures. There are rare reports of neonates with HCMV infection who developed hepatic fibrosis, non-cirrhotic portal hypertension, giant-cell hepatitis or intra-hepatic calcification [4, 11-17]. Acute infection of either type 1 or 2 herpes simplex virus (HSV) may have a role in liver failure of newborns. HSV nuclear inclusions were detected in liver biopsy specimens of infected neonates with areas of necrosis [4-7]. From hepatotropic viruses, the highest risks belong prenatal transmission of hepatitis C virus (HCV) and especially hepatitis B virus (HBV) infections in neonates. Acute neonatal hepatic failure or severe hepatitis after exposure to these viruses are generally presented in subclinical form and rarely seen in the neonatal period [4, 18, 19]. The possible role of rotavirus infection in induction of neonatal hepatitis is attributed to the high infantile prevalence of this viral infection, its important role in neonatal diarrhea, and its familiar relationships with serotype 3 of reovirus as one of the widely discussed viral inducers of neonatal cholestasis [20]. Different types of adenoviruses can rarely infect neonatal liver. In some case studies, it was shown that adenovirus-related hepatitis with a mortality rate of almost 80% may occur in the first two weeks of life [3, 4]. Therefore, in this research we studied the tissue impact of HCMV, HSV, HBV, HCV, rotavirus and adenovirus infections on infants with neonatal hepatitis.

## PATIENTS AND METHODS

After clinical and pathological exclusion of other types of hepatitis disorders, 22 infants who suffered from neonatal hepatitis, admitted from 1996–2007 to Organ Transplant Unit, Namazee Hospital, Shiraz University of Medical Sciences, Shiraz, southern Iran, were included in this cross-sectional study. The paraffin-embedded biopsy and autopsy tissues of these infants were retrieved from the pathology file of the laboratory of Namazee Hospital, cut to small sections, and stored at room temperature until molecular and antigenic virology tests were preformed. The molecular and antigenic assays were performed double blind for elimination of any possible technologist errors.

Definition of Neonatal Hepatitis

Neonatal hepatitis refers to a group of liver dysfunctions that affect infants between the ages of about one and two months, which for decreasing bile flow also produce a typical jaundice. Infants with neonatal hepatitis have normal, intact, bile ducts. Strictly speaking, this implies elevated serum bile acids, but in practice, it is usually defined by the presence of conjugated hyperbilirubinemia [3]. The hallmark of findings in the liver of many newborns with neonatal hepatitis is giant-cell hepatitis characterized by inflammation and large multinucleated hepatocytes in the liver parenchyma. Structural disorders causing obstruction of large bile ducts lead to typical features of duct obstruction in portal tracts and surrounding parenchyma. Typical changes of various metabolic diseases may be evident on liver biopsy in the affected infants [4].

Liver Demographic Data

The liver-related hematological and biochemical laboratory records including blood urea nitrogen (BUN), white blood cell (WBC) count, red blood cell (RBC) count, aspartate aminotransferase (AST), alanine aminotransferase (ALT), alkaline phosphatase (ALP), direct and total bilirubin (D-Bili and T-Bili), albumin (Alb), and globulin (Glu) were analyzed in this study for all studied neonates. 

Viral Molecular Assays

*Viral genome extraction*

The DNA genome of HCMV, HSV, HBV, and adenovirus was extracted from the paraffin-embedded biopsy and autopsy specimens (QIAamp DNA Mini kit, QIAGEN, Germany) according to the manufacturer’s instruction. Also, the RNA genome of HCV and rotavirus was extracted form the specimens (QIAamp RNA Mini kit, QIAGEN, Germany).

*PCR protocols of DNA viruses*

An in-house qualitative PCR protocol was optimized for the diagnosis of adenovirus infection in tissue samples of newborns with neonatal hepatitis with standard and confirmed negative and positive controls. The presence of adenovirus DNA genome was evaluated by primer pair that amplified a 81-bp fragment of gene II of adenovirus which is highly conserved among different serotypes with newly designed primers sequences; the forward primer: 5^′^-TTC CTC TAT CTC AGA CAC TGG CTC A-3^′^; and reverse primer: 5^′^-CCA AGC GGC CTC TGA TAA CCA-3^′^. The mixture condition of PCR reaction in a total volume of 50 μL contains 5 μL of 10X PCR buffer, 1 μL MgCl_2 _(50 mM), 1.5 μL dNTP (10 mM), 1 μL of each primer (20 pmol), 0.5 μL Taq (2.5 unit), and 5 μL of the sample DNA. Use of 94 °C for 3 min, and run a program including 35 cycles of 1 min at 94 °C, 1 min at 62 °C and 1 min at 72 °C and finally, one cycle of 5 min at 72°C is the thermocycling condition of this PCR technique.

HCMV genome was detected in paraffin-embedded tissue samples by an in-house nested-PCR protocol that optimized by standard and confirmed negative and positive controls. The outer primers sequences are: forward primer: 5^′^-TAC TGC ACG TAC GAG CTG TT-3^′^ and reverse primer: 5^′^-GCG TAC GTG ATG AGG CTA TAA-3^′^ amplify a 600-bp sequence of HCMV-UL55 gB encoding sequence. The mixture condition of PCR reaction in a total volume of 50 μL containing 5 μL of 10X PCR buffer, 1.5 μL of MgCl_2 _(50 mM), 1 μL dNTP (10 mM), 1 μL of each primer (10 pmol), 0.5 μL Taq (2.5 unit), and 5 μL of the sample DNA. Theromocycling conditions of the first round amplification of HCMV genome were as follows: First denaturation step 93 °C for 3 min, continuing with a program including 30 cycles of 1 min at 93 °C, 1 min at 60 °C and 1 min at 72 °C; and finally, one cycle of 5 min at 72 °C. The second round of HCMV nested-PCR was optimized with inner primers sequences of: forward primer: 5^′^-CCT TCA CGT TCA TAT CAC GC-3^′^ and reverse primer: 5^′^-GTG GAA CTG GAA CGT TTG GC-3^′^ that amplify a 384-bp sequence of HCMV-UL55 gB encoding sequence. Five μL of the first round PCR product was included in the second round PCR mixture which was the same as the first round PCR mixture. The first denaturation step was at 94 °C for 3 min, continuing with a program including 30 cycles of 1 min at 93 °C, 1 min at 59 °C and 1 min at 72 °C; and finally, one cycle of 5 min at 72 °C. Based on the manufacturer’s guidelines, detection of HBV-DNA and HSV-DNA were performed by qualitative HBV-PCR kit (Cinnagen, Iran) and by qualitative HSV-PCR kit (Sacace, Italy), respectively.

*RT-PCR protocols RNA viruses*

In tissue samples of infants with neonatal hepatitis, HCV-RNA and the genome of rotavirus were diagnosed by nested HCV-RT-PCR kit (Cinnagen, Iran) and by qualitative RT-PCR kit (Sacace, Italy), according to the manufacturer’s protocols, respectively.

*IHC protocol*

Detection of HCMV, HSV, HBV, and adenovirus proteins in paraffin-embedded tissue sections of neonates with hepatitis was performed by immunohistochemistry (IHC) method that is accomplished with highly specific monoclonal antibodies as follow: Serial sections with 3 μm thickness were obtained from each specimen and mounted on poly-L lysine coated slides for IHC evaluation. IHC was performed on selected representative paraffin blocks of each case by using streptavidin-biotin peroxidase complex detection system (LSAB kit, Dako, Denmark) with the presence of adequate and appropriate positive and negative controls. For negative controls, all steps of the procedure were performed without primary antibodies. In this study, HBV antigens including HBsAg and HBcAg were diagnosed in these tissue sections by anti-HBs mAb and anti-HBc mAb (Dako, Denmark), respectively. Adenovirus hexon proteins, and HSV-1 and 2 antigens were detected in paraffin-embedded tissue sections by specific monoclonal antibodies (Dako, Denmark). Also HCMV antigen was diagnosed in these biopsy and autopsy tissue sections by a specific monoclonal antibody (Signet, USA). 

## RESULTS

Of the 22 studied neonates with hepatitis disorders, 19 (86%) were male and three (14%) were female. The age of patients ranged from 3 to 120 (mean: 47) days. One of these infants, died of disseminated intravascular coagulation. Mild fibrosis was diagnosed in three (14%) of 22 studied infants. The molecular and antigenic etiology of viral infections and also the laboratory and clinical findings of the studied infants are presented in Tables 1 and 2.

**Table 1:** The molecular and antigenic etiology of viral infections in patients with neonatal hepatitis

Patients (No.)	Age (days)	Sex	HCMV-DNA	Rotavirus-DNA	ADV-DNA	HCV-RNA	HSV-DNA	HBV-DNA	HSV-1 Ag	HSV-2 Ag	HCMV-Ag	HBV-HBsAg	HBV-HBcAg	ADV-Ag
1	3	F	–	–	–	–	–	+	–	+	–	–	–	–
2	60	M	–	+	–	–	–	+	–	–	–	–	+	–
3	60	M	–	–	–	–	–	–	–	+	–	–	–	–
4	60	M	–	–	–	+	–	–	–	+	–	–	–	–
5	60	M	–	–	–	–	+	–	–	–	–	–	–	–
6	60	M	–	–	–	–	–	–	–	–	–	–	–	–
7	45	M	–	–	–	–	–	–	–	+	–	–	–	–
8	12	M	–	–	–	–	–	–	–	–	–	–	–	–
9	60	M	+	–	–	–	+	–	–	–	–	–	–	–
10	90	M	–	–	–	–	–	–	–	–	–	–	–	–
11	60	M	–	–	–	–	–	–	–	–	–	–	–	–
12	90	M	–	–	–	–	–	–	–	–	–	–	–	–
13	30	F	–	–	–	–	+	–	–	–	–	–	–	–
14	60	M	–	–	–	–	–	–	–	–	–	–	–	–
15	90	M	–	–	–	–	–	–	–	–	–	–	–	–
16	3	F	–	–	–	–	–	–	–	–	–	–	–	–
17	60	M	–	–	–	–	–	–	–	–	–	–	–	–
18	120	M	–	–	–	–	–	–	–	–	–	–	–	–
19	60	M	–	–	–	–	–	–	–	–	–	–	–	–
20	60	M	–	–	–	–	–	+	–	+	–	–	–	–
21	30	M	–	–	–	–	–	+	–	–	–	–	–	–
22	60	M	–	–	–	–	–	–	–	–	–	–	–	–

M: Male; F: Female

HCV infection and Neonatal Hepatitis

Only one patient was found positive for HCV infection by molecular methods (Table 1).

Rotavirus infection and Neonatal Hepatitis

The RNA genome of rotavirus was detected in biopsy section of one newborn with hepatitis (Table 1).

HBV infection and Neonatal Hepatitis 

The HBV genome was detected in 3 (14%) of 22 liver biopsy and autopsy specimens. HBcAg was only detected in one tissue sample of studied infants with neonatal hepatitis infected with HBV-DNA (Table 1). 

HSV infection and Neonatal Hepatitis 

Three (14%) of 22 studied neonates was infected with HSV-DNA. The HSV-1 specific antigen was detected in only one of these three HSV-DNA infected tissues; HSV-2 specific antigen was diagnosed in five (23%) of 22 liver autopsy and biopsy specimens. However, none of the HSV-2 antigen positive samples were found positive for HSV-DNA infection. Mild liver fibrosis was seen in one of HSV-DNA infected patients. One of HSV-2 antigen infected newborns was also suffered from G6PD deficiency (Table 1).

HCMV infection and Neonatal Hepatitis 

The genome of HCMV was found in the liver biopsy and autopsy section of only one patient. HCMV antigen was detected in none of liver tissue sections studied (Table 1). 

Adenovirus infection and Neonatal Hepatitis

The genome and antigen of adenovirus was not diagnosed in liver autopsy and biopsy tissue sections of studied neonates (Table 1). 

Viral co-infections and Neonatal Hepatitis

Some of the studied newborns were infected with more than one viral infection. HBV-DNA and HSV-2 antigen were detected simultaneously in one newborn with high BUN level who ultimately died of disseminated intravascular coagulation. Another neonate with hepatitis was had HCMV-DNA, HSV-DNA, and HSV-1 antigen. The HCV molecular infection and also the antigen of HSV-2 were detected simultaneously in another patient. Finally, one patient had HBV-DNA, HBcAg and rotavirus genome (Table 1).

Liver indices and Neonatal Hepatitis

The level of AST, ALT, and ALK was increased in 100%, 86%, and 52% of the studied neonates, respectively. The rise in BUN, Alb, and Glu level was found less frequently in 5%, 10%, and 5% of studied newborns, respectively. The level of ALT, AST, ALK, T-Bili, D-Bili, was highly increased in the patient infected with HCMV, HBV, HCV, HSV, rotavirus, and adenovirus. The Alb level was normal during neonatal hepatitis in viral infected patients (Table 2). In 19% of studied patients with viral infection, the WBC count was increased.

## DISCUSSION

Despite extensive evaluation of clinical, pathological, and laboratory findings related to neonatal hepatitis, the cause of this disorder in majority of infants remains unknown [21]. Neonatal hepatitis may be caused by different underlying conditions including microbial infections, anatomic abnormalities, metabolic disorders, genetic malfunctions, neoplastic diseases, vascular erosions, toxic agents, dysfunction of immune system, and other rare disease conditions that impair the normal liver function [4]. Many cases, however, seem to occur for no apparent reason (sporadic). Viruses have an important role in the pathogenesis of this type of newborn liver failure. Hepatotropic and non-hepatotropic viruses are among the most important microbial inducers of neonatal hepatitis [10]. Among the hepatotropic viral infectious agents, HBV for its widespread induction of prenatal acute infection is of particular importance for neonatal hepatology investigators [22, 23]. But HBV-related hepatic failure may rarely occur in neonates [3, 14, 19]. In this study, three neonates were found positive for HBV-DNA. In one of these infants, the tissue presentation of HBcAg and simultaneous co-infection with rotavirus were confirmed. Also, co-presence of HSV-2 antigen was seen in another HBV-infected neonate. Similar to these findings, Livramento, *et al.*, reported HBV infection in 2.2% of patients with neonatal hepatitis [24]. But Amer, *et al.*, could not detect HBV infection (HBsAg) in the sera of neonates with cholastasis [7]. In comparison to infantile HBV infections, HCV mostly causes chronic hepatitis that explains the lower importance of this viral infection in the pathogenesis of neonatal hepatitis. The molecular diagnosis of HCV infection was confirmed in only one of the studied neonates with hepatitis who was also found positive for HSV-2 antigen. Similar to these results, in another study, 81% of the infants perinatally acquired HCV infection, were asymptomatic for their chronic progressive hepatitis [25].

From non-hepatotropic viral infectious agents of infantile hepatitis, the pathogenic role of herpes virus for its ubiquitous vertical transmission, is more critical. The infection of HCMV genome was confirmed in liver tissue of only one infant with neonatal hepatitis. This result is in accord with other research studies. Prado, *et al.*, found HCMV infection in only 2.2% of infants with cholestasis [26]; Goedhals, *et al.*, also detected HCMV infection in only 1 of 85 infants with jaundice [17]; and Lurie, *et al.*, could not detect any cytomegalic cells in 84 infants with hepatitis [27, 28]. On the other hand, many investigators stressed on the important role of HCMV infection in neonatal hepatitis. Chang, *et al.*, found HCMV-DNA in 46% of infants with neonatal hepatitis [11]. Also, Fischler, *et al.*, in Sweden infants with cholestasis, and Oliveira, *et al.* in 76 Brazilian neonates with cholestasis, diagnosed high prevalence of HCMV infection [29, 30].

Both HSV-1 and HSV-2 may relate to the pathogenesis of neonatal hepatitis [4]. Hepatic pathology resulted from HSV infection is characterized by irregular areas of necrosis with intra-nuclear eosinophilic and/or amphophilic viral inclusions [31]. In this research, HSV genome infection was seen in 3 (14%) of 22 studied neonates. The antigen of HSV-1 was detected in only one of these three patients; HSV-2 antigen was detected in 5 (23%) of 22 studied newborns. But, none of those with HSV-2 antigen were positive for HSV-DNA simultaneously. In accord to these results, some authors suggested a link between HSV infection and neonatal cholestasis [30, 32, 33]. But, they stated that HSV infection has no role in the pathogenesis of neonatal cholestasis. Simultaneous co-infection with HCMV and HSV was seen in one newborn with hepatitis.

Multi-organ replication of adenovirus was reported in some case studies including the rare report of adenoviral hepatitis and disseminated intravascular coagulation with a mortality approaching 80% and its commonly occurrence in newborns [3, 4]. But in this study, the molecular and antigenic determination of adenoviruses was not seen in any liver autopsy and biopsy tissue specimens of the studied neonates.

The infective role of reoviruses and with a lower attention rotaviruses as RNA viral infectious agents that may have role in the pathogenesis of neonatal hepatitis was studied well and controversially confirmed by other researchers [20, 32]. Rotavirus is an important viral causes of microbial-related gastrointestinal disorders in human newborns and like reoviral infections may also cause neonatal hepatitis. In this study, rotavirus genome was detected in only one newborn who also had the HBV genome and antigen. This result was also found in other studies. Richardson, *et al.* reported a 50% seroprevalence of Reo type III in neonates with cholestasis [34]. This association was however not observed in another study [26].
